# Supplementary material for: Homologous Ad26.COV2.S vaccination results in reduced boosting of humoral responses in hybrid immunity, but elicits antibodies of similar magnitude regardless of prior infection
Source: medRxiv. 2023 Mar 15:2023.03.15.23287288. Preprint. [Version 1] doi: 10.1101/2023.03.15.23287288 (PMC10055608; doi:10.1101/2023.03.15.23287288)
Supplement: Supplement 1 — Supplementary Figure 1. Study design and participant demographics. (A and B) Schematic representing the study design and participant demographics. Participants were categorized into three groups: Group 1: no infection prior to vaccination (n=13), Group 2: D614G-infected prior to vaccination (n=14) and Group 3: Beta-infected prior to vaccination (n=16). All three groups were followed for 6 months after initial vaccination. A subset of n=6 Group 1, n=10 Group 2 and n=8 Group 3 participants received a homologous Ad26.COV2.S vaccination and boosting effect was analyzed after 1 month. Schematic was created using BioRender.com. Supplementary Figure 2. Flow cytometry gating strategy. Gating strategy (A and B) and representative examples of SARS-CoV-2 spike-specific IFN-γ, IL-2 and TNF-α production in memory CD4+ and CD8+ T cells (C). Representative plots showing spike-specific T cell immune phenotyping (D). ED: early differentiated, LD: late differentiated, Eff: effector, Inter: intermediate. Supplementary Figure 3. Durability of binding responses 6 months after a single dose of Ad26.COV2.S. Plasma samples from participants with no prior infection (black), infected with D614G (blue) or infected with Beta (red) were tested for binding responses to the Beta (A), Delta (B) and BA.1 (C) variants at three time points (pre-vaccination, 1 and 6 months post-vaccination). Geometric mean values and median fold changes are shown below the graphs. Fold changes were all calculated relative to the pre-vaccination time points. Binding antibodies were quantified by OD450nm values. All experiments were performed in duplicate. The Friedman test with Dunn’s correction for multiple comparisons was used to determine statistical significance. Significance is shown as: ****p<0.0001, ***<0.001, **p<0.01, *p<0.05. Supplementary Figure 4. Durability of antibody dependent cellular cytotoxicity 6 months after a single dose of Ad26.COV2.S. Plasma samples from participants with no prior infection (blac [file media-1.pdf]

A

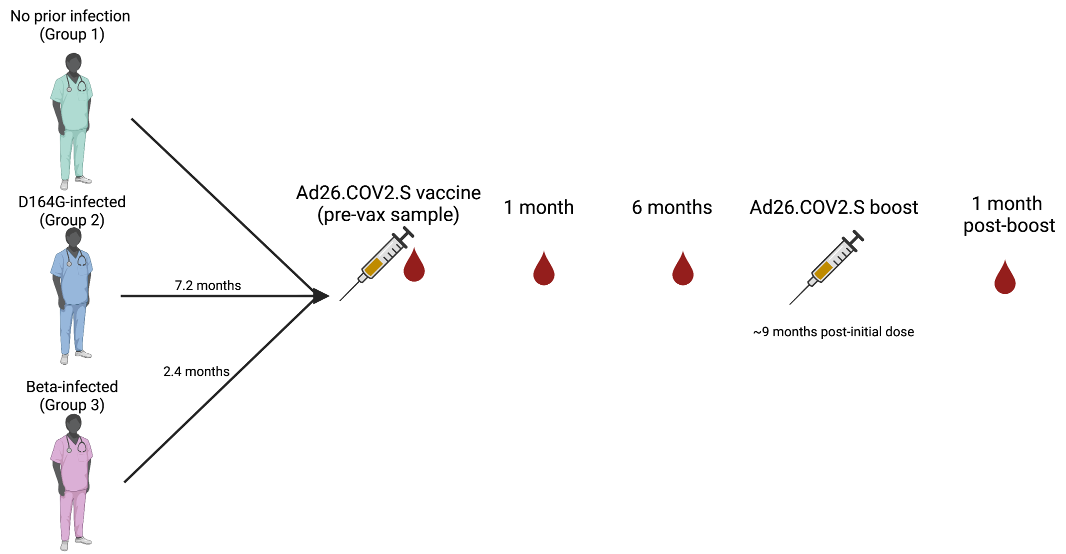

B

	Longitudinal cohort		
	No prior infection	D614G infected	Beta infected
N	13	14	16
Age (median, IQR)	54 [35-58]	35 [29-37]	38 [33-48]
Gender (% female)	77%	64%	75%
Time after initial vaccine dose (months, IQR)	5.9 [5-6.3]	5.3 [5.1-6.2]	5.4 [5.1-6.1]

Supplementary Figure 1

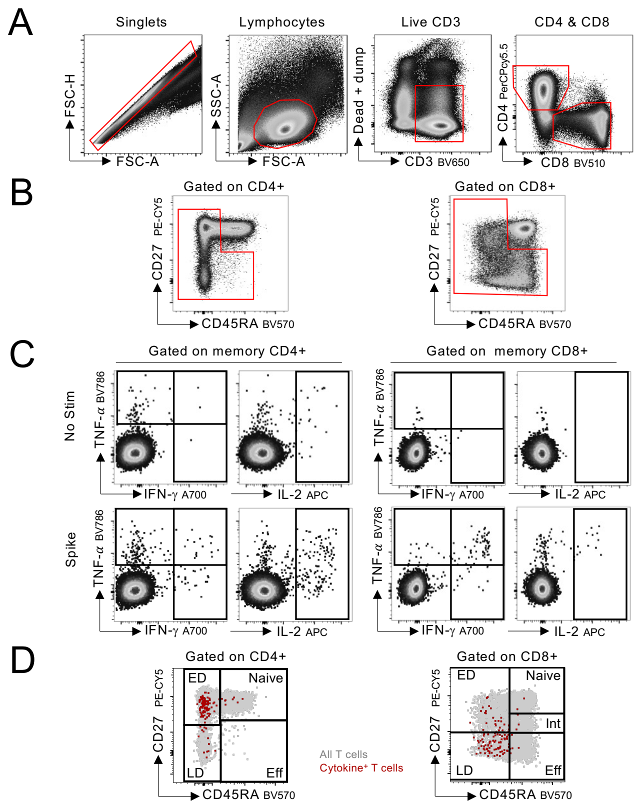

Supplementary Figure 2

Binding

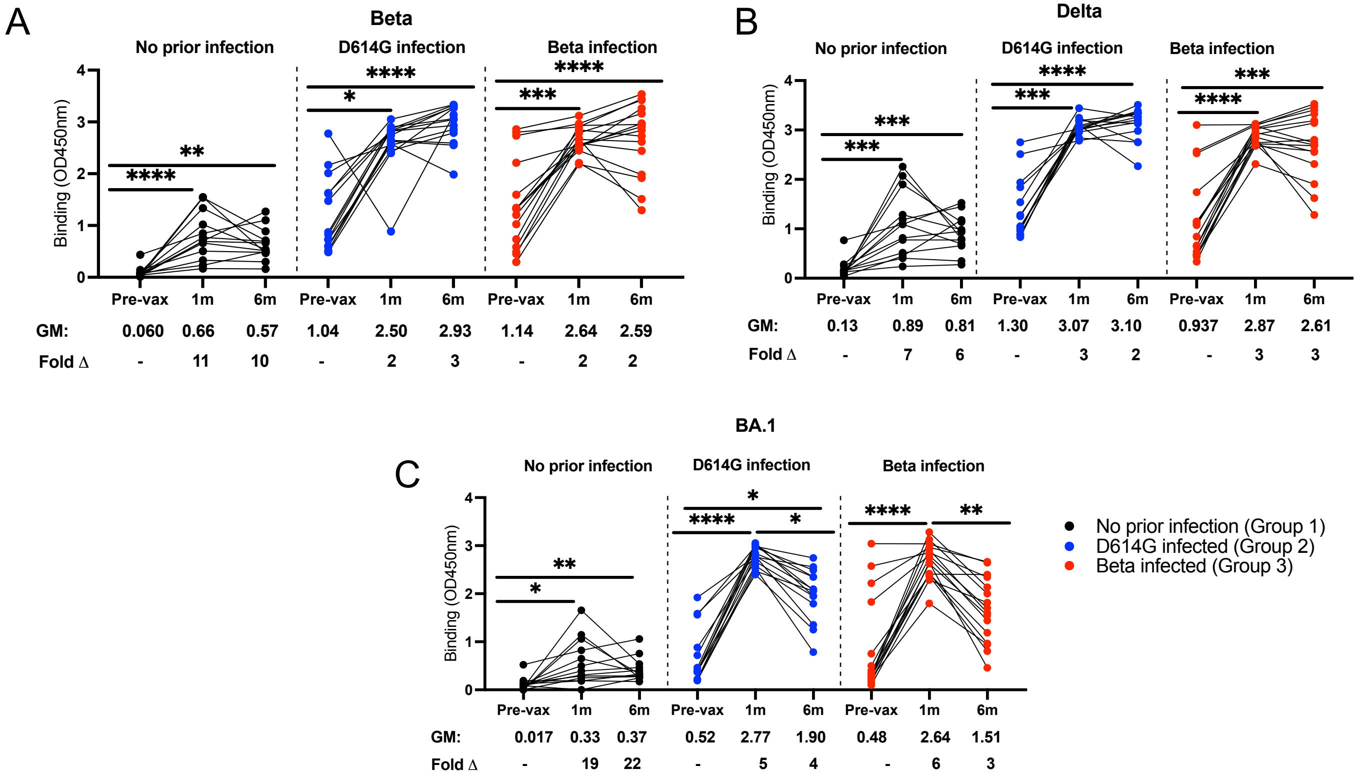

Supplementary Figure 3

Antibody dependent cellular cytotoxicity (ADCC)

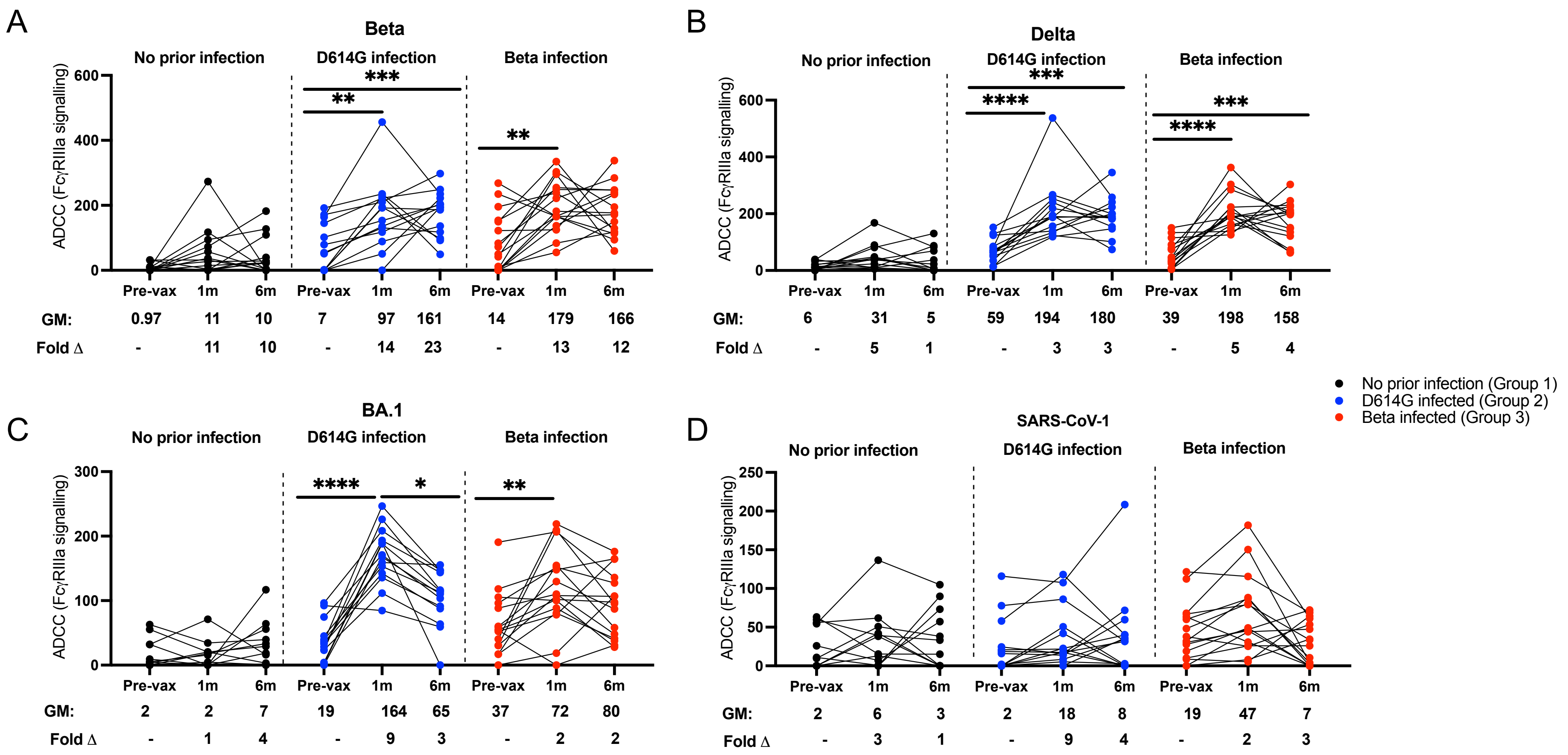

Supplementary Figure 4

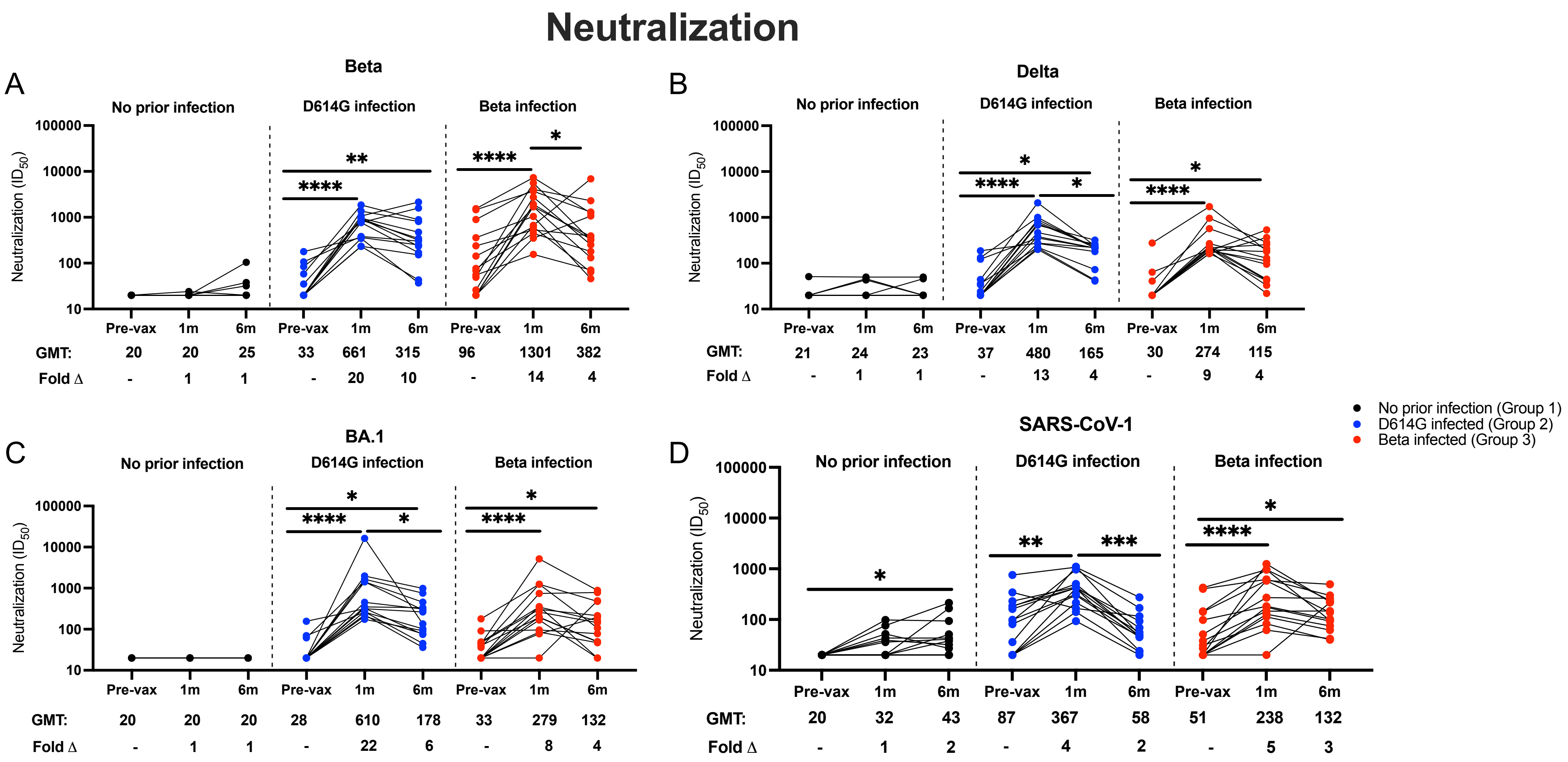

Supplementary Figure 5
